# Comparative analysis of gut microbiota in metabolic syndrome and obese children from Southeastern China

**DOI:** 10.3389/fmicb.2024.1503302

**Published:** 2024-12-23

**Authors:** Jingjing Wang, Peifeng Zhuang, Bin Lin, Jinlu Zheng, Haiqing Li, Wenlin Tang, Wenbin Ye, Xiangjian Chen, Mingping Zheng

**Affiliations:** ^1^Ningde Municipal Hospital, Ningde Clinical Medical College of Fujian Medical University, Fuzhou, China; ^2^Department of Pediatrics, Ningde Municipal Hospital of Ningde Normal University, Ningde, China; ^3^Department of Joint Surgery and Sports Medicine, Ningde Municipal Hospital of Ningde Normal University, Ningde, China

**Keywords:** gut microbiota, metabolic syndrome, obese children, weight, fecal microbiota transplantation (FMT)

## Abstract

The prevalence of childhood obesity is rising globally, with some obese children progressing to develop metabolic syndrome (MS). However, the specific differences between these groups remain unclear. To investigate the differences in gut microbiota, we conducted physiological and biochemical assessments, alongside 16S rRNA sequencing, in a cohort of 32 children from Southeastern China, which included 4 normal-weight children, 5 with mild obesity, 9 with moderate obesity, 9 with severe obesity, and 5 with metabolic syndrome. Our results indicated that waist circumference, serum triglycerides, total cholesterol, non-HDL levels, and the prevalence of fatty liver were significantly elevated in both obese and MS children compared to their normal-weight peers, with the MS group exhibiting more pronounced abnormalities. Conversely, HDL levels showed a contrasting trend. Additionally, alpha diversity of gut microbiota increased with weight, while beta diversity analysis revealed significant compositional differences between children with MS and those who were normal weight or obese. At the class and genus levels, we found that the relative abundance of c_Gammaproteobacteria increased with weight, whereas c_Bacteroidia and g_Bacteroides decreased. Notably, g_Faecalibacterium was significantly less abundant in the MS group compared to the other cohorts. LEfSe and functional analyses identified distinct gut microbiota and functional differences between children with MS and those with normal weight or obesity. Furthermore, gavage experiments in mice showed that gut microbiota from obese and MS subjects significantly increased serum triglycerides and cholesterol levels, leading to hepatocellular damage. In contrast, fecal gavage from normal-weight individuals into obese model mice significantly reduced serum triglycerides and the number of degenerative liver cells, as well as the extent of fat accumulation. These findings provide critical insights into the understanding and management of obesity and metabolic syndrome in pediatric populations.

## Introduction

Obesity, defined as an excess of adipose tissue, is widespread globally and affects individuals of all age groups (NCD Risk Factor Collaboration (NCD-RisC), 2017). In recent years, the evolution of the global economy, coupled with changes in dietary habits and other factors, has led to a significant increase in the prevalence of childhood obesity worldwide. Between 1975 and 2016, the prevalence of overweight and obesity among children and adolescents aged 5 to 19 rose from 4% to over 18% (Zhang and Ma, 2018). According to the World Obesity Federation’s Childhood Obesity Atlas Report in 2019, it is projected that approximately 254 million children and adolescents globally, aged 5 to 19 years, will be affected by obesity by 2030. Obesity has led to the emergence of multiple serious obesity-related comorbidities (Dietz and Robinson, 2005) and has the potential to adversely affect virtually every system within the human body (Skinner et al., 2015; Zuñiga Vinueza and Jaramillo, 2023). Children with obesity are at an increased risk for hyperinsulinemia (Molnár, 2004), musculoskeletal problems (Chan and Chen, 2009), idiopathic intracranial hypertension (Apperley et al., 2022), and other diseases. Thus, obesity has escalated into one of the most pressing public health crises of our time.

Metabolic syndrome is characterized as a constellation of cardio-metabolic risk factors that predispose individuals to cardiovascular diseases and type 2 diabetes mellitus. The key components include central obesity, indicated by elevated waist circumference (WC), dysglycemia/insulin resistance (IR), hypertension, hypertriglyceridemia, and reduced levels of high-density lipoproteins (HDL) (Alberti et al., 2009). The pathogenesis of metabolic syndrome is intricate, with many aspects still not fully elucidated (Al-Hamad and Raman, 2017; Vanlancker et al., 2017). It is postulated that central obesity and/or IR activate multiple pathogenic pathways that enhance metabolic risk, culminating in the full manifestation of the syndrome (Alberti et al., 2009; Johnson et al., 2009). In light of the increasing prevalence of childhood metabolic syndrome, it is anticipated that cardio-metabolic abnormalities will also become more prevalent among youth (Miller et al., 2014). The early emergence of risk factor clustering is particularly concerning, as the components of metabolic syndrome may persist into adulthood, significantly elevating the risk for future type 2 diabetes mellitus and cardiovascular disease (Mameli et al., 2017).

The gut microbiota is the most diverse microbial community within the human body. Through a process of long-term co-evolution, it has established a symbiotic relationship with the host, significantly influencing gene expression, gut barrier integrity, nutrition, metabolism, and overall immune function (Liu et al., 2017). This microbiota is essential for maintaining homeostasis and is often described as a “second genome,” particularly in the context of metabolic disease development. As such, gut microbiota-targeted interventions, including probiotics, prebiotics, fecal microbiota transplantation, metabolic surgery, and pharmacological agents, may serve as effective strategies for mitigating obesity and metabolic syndrome. Recent research has identified gut microbiota dysbiosis as a contributing risk factor for the onset of obesity and metabolic syndrome (Chen et al., 2020; Wang et al., 2024; Carrizales-Sánchez et al., 2021; Carrizales-Sánchez et al., 2023). However, there remains a significant gap in the literature regarding the compositional differences in gut microbiota between obese children and those with metabolic syndrome.

To investigate the differences in gut microbiota between obese children and those with metabolic syndrome, we conducted physiological and biochemical assessments, along with 16S rRNA sequencing, in a cohort of 32 children from Southeastern China, encompassing individuals with mild obesity, moderate obesity, severe obesity and metabolic syndrome. Our findings provide critical insights and a foundational basis for the understanding and management of obesity and metabolic syndrome in pediatric populations.

## Materials and methods

### Study cohort and sample collection

In the period from March to July 2023, an epidemiological survey of obese children was conducted in four schools (two elementary and two middle schools) within the central urban area of Ningde, following approval from the Ningde City Education Bureau and obtaining informed consent from the guardians of the children. The survey identified children aged 6 to 16 who met the diagnostic criteria for obesity. Additionally, normal weight children were selected from the same schools for comparative analysis.

All children were screened to exclude other diseases, including renal, endocrine, genetic metabolic, central nervous system disorders, chromosomal abnormalities, and genetic conditions. The diagnostic criteria for obesity and overweight were based on the “Expert Consensus on the Diagnosis, Assessment, and Management of Childhood Obesity” established by the Endocrinology, Genetics, and Metabolism Group of the Chinese Pediatric Society in 2022. According to the “Expert Consensus on Medical Nutritional Therapy for Overweight/Obesity (2016 Edition),” the World Health Organization recommends assessing obesity in children using the height-based standard weight method. For children of the same height with adequate nutrition, the standard weight is defined as 100%, with a range of ±10% considered normal. A weight exceeding 15% above the standard is classified as overweight, greater than 20% as mild obesity, greater than 30% as moderate obesity, and greater than 50% as severe obesity. The diagnostic criteria for metabolic syndrome in children and adolescents were based on the “Definition and Prevention Recommendations for Childhood and Adolescent Metabolic Syndrome” by the Chinese Pediatric Society in 2012. Prior to fecal sample collection, participants were required to have not taken antibiotics for 2 weeks, be free from stress, have no history of vaccination, and show no gastrointestinal symptoms such as constipation or diarrhea. A total of 32 children participated in this study, including 4 normal-weight children, 5 with mild obesity, 9 with moderate obesity, 5 with severe obesity, and 5 with metabolic syndrome. Fecal samples from all participants were collected in sterile containers and frozen at −20°C within 1 h after collection.

The studies involving human/animal participants were reviewed and approved by Ningde Municipal Hospital of Ningde Normal University, and the approval number is 20220810.

### DNA extraction, PCR amplification, and sequencing

Total bacterial DNA was extracted from fecal samples using the E.Z.N.A. Mag-Bind Soil DNA Kit. The concentration and purity of the DNA were assessed using the Qubit 3.0 DNA detection kit. The quality of the extracted DNA from fecal microbiota of children with varying weights was verified through 1% agarose gel electrophoresis. Primers targeting the 16S V3–V4 region (341F 5’-CCTACGGGNGGCWGCAG-3′, 806R 5’-ACTACNVGGGTWTCTAAT-3′) were used for amplification of the extracted microbial DNA. All primers contained an 8-nucleotide barcode sequence unique to each sample. Then the PCR products were recovered using 2% agarose gel electrophoresis, and purified by Agencourt AMPure XP kit. The concentration of the recovered products was measured with the Qubit 3.0 fluorometer, and sequencing was performed on the Illumina NovaSeq 6,000 platform. Raw data can be accessed from NCBI SRA (Bioproject ID: PRJNA1165468).

### Data processing

Paired-end reads was assigned to samples based on their unique barcodes and truncated by cutting off the barcodes and primer sequences. The whole process was performed through Python (V3.6.13) and adaptors were removed through cutadapt (V3.3). Paired-end reads were merged using FLASH (V1.2.11, http://ccb.jhu.edu/software/FLASH/) (Magoc and Salzberg, 2011), and the splicing sequences were called raw tags. Quality filtering on the raw tags were performed using the fastp (Version 0.23.1) software to obtain high-quality Clean Tags (Bokulich et al., 2012). And the effective tags were obtained by removing the chimera sequences with the vsearch (V2.16.0) package (Edgar et al., 2011). Sequences analysis were performed by Uparse software (Uparse v7.0.1001, http://drive5.com/uparse/) (Edgar, 2013). Sequences with ≥97% similarity were assigned to the same OTUs. Representative sequence for each OTU was screened for further annotation. For each representative sequence, the Silva Database^1^ (Quast et al., 2012) was used based on Mothur algorithm to annotate taxonomic information. Alpha diversity were calculated with QIIME (Version 1.9.1) and displayed with R software (Version 4.0.3). Beta diversity on both weighted and unweighted unifrac were calculated by QIIME software (Version 1.9.1). Top 10 taxa or 25 taxa of each samples at each taxonomic ranks (Phylum, Class, and Genus) were selected to plot the distribution histogram of relative abundance in Perl through SVG function. Effect Size analysis was applied with LEfSe software (Segata et al., 2011). LDA (LDA score ≥ 3) was used to estimate the effect size of each taxon differentially represented in cases and controls. Tax4Fun (V0.3.1) was used to predicted metabolic pathway.

### Statistical analysis

Statistical analyses were performed depending on the normality of the data, assessed using the ANOVA and t-test. Statistical significance was determined at an alpha level of *p* < 0.05.

### Mouse husbandry and handling for fecal transplantation from children of different weights

Six-week-old male C57BL/6 mice were acquired from Beijing Sibef Biotechnology Co., Ltd. The mice were housed under controlled conditions with a temperature of 20–26°C and humidity levels maintained at 40–70%, with ad libitum access to water and food. After a one-week acclimatization period, the mice were randomly assigned to four experimental groups: the saline gavage group, the normal children’s feces gavage group, the feces from children with metabolic syndrome gavage group, and the feces from obese children gavage group. Fecal samples were collected and dissolved in 0.9% NaCl at a ratio of 1 g:10 mL, followed by homogenization for 5 min. Subsequently, 0.1 mL of 10% glycerol was added to the homogenate, which was then administered via gavage at a dose of 1 mL/100 g once daily for 6 consecutive weeks. Prior to fecal administration, all mice received an antibiotic regimen consisting of metronidazole (1 g/L), vancomycin (1 g/L), and streptomycin (2 g/L), administered once daily for 3 days.

### Development and handling of obesity model mice following fecal and saline intragastric treatment

The mice underwent a one-week acclimatization period and were subsequently randomized into a control group and an obesity model group. The control group received a standard diet, while the obesity model group was subjected to a high-fat diet for eight consecutive weeks to establish the obesity model. After the modeling phase, the obese mice were randomly allocated to two treatment groups: one receiving gastric gavage of physiological saline and the other receiving gastric gavage of feces from normal-weight children. Fecal samples were processed by dissolving them in 0.9% NaCl at a ratio of 1 g:10 mL, followed by homogenization for 5 min. To this solution, 0.1 mL of 10% glycerol was added, and the resulting mixture was administered via gavage at a dosage of 1 mL/100 g, once daily for 6 weeks. Prior to fecal gavage, all animals were treated with a combination of antibiotics (metronidazole at 1 g/L, vancomycin at 1 g/L, and streptomycin at 2 g/L) via gavage, once daily for three consecutive days.

### Biochemical analyses of triglyceride and cholesterol levels

Serum was collected from the mice, and triglyceride (TG) levels were measured using a fully automated biochemical analyzer (BK-600, Shandong Bokang) with the assay kit (70,102, Shandong Bokang Biotech). Cholesterol (CHO) levels were assessed using the corresponding kit (701,132, Shandong Bokang Biotech). The analyses were conducted according to the manufacturer’s instructions.

### HE staining for hepatic pathology

Liver tissue samples from mice were prepared as paraffin sections and subjected to baking, deparaffinization, and rehydration. The sections were stained with hematoxylin for 3–5 min, followed by rinsing in running water. Differentiation was performed using 1% hydrochloric acid in ethanol, and counterstaining was conducted with eosin for 3–5 min. After dehydration, the sections were mounted for examination under a microscope (BX43, Olympus).

### Oil Red O staining for hepatic lipid accumulation

Frozen sections were fixed in 4% paraformaldehyde for 10 min and subsequently washed thoroughly with distilled water. The sections were then treated with 60% isopropanol for 2 min, followed by staining with Oil Red O for 10 min. Rapid differentiation was performed using 60% isopropanol, and the sections were washed with distilled water. They were then stained with hematoxylin for 3 min, differentiated using hydrochloric acid in ethanol for 10 s, briefly rinsed, and subjected to a bluing solution for 15 s. After a final wash with running water, the sections were photographed for analysis.

## Results

### Physiological and biochemical indicators of normal weight, obese, and metabolic syndrome children

To examine the differences in physiological and biochemical indicators among children with varying obesity levels, those with metabolic syndrome, and normal-weight children, we assessed 32 participants. This included 4 normal-weight children, 5 with mild obesity, 9 with moderate obesity, 9 with severe obesity, and 5 with metabolic syndrome. We measured waist circumference, blood pressure, triglycerides, cholesterol, high-density lipoprotein (HDL), non-high-density lipoprotein (non-HDL), liver function, and fatty liver prevalence.

**Figure 1:**
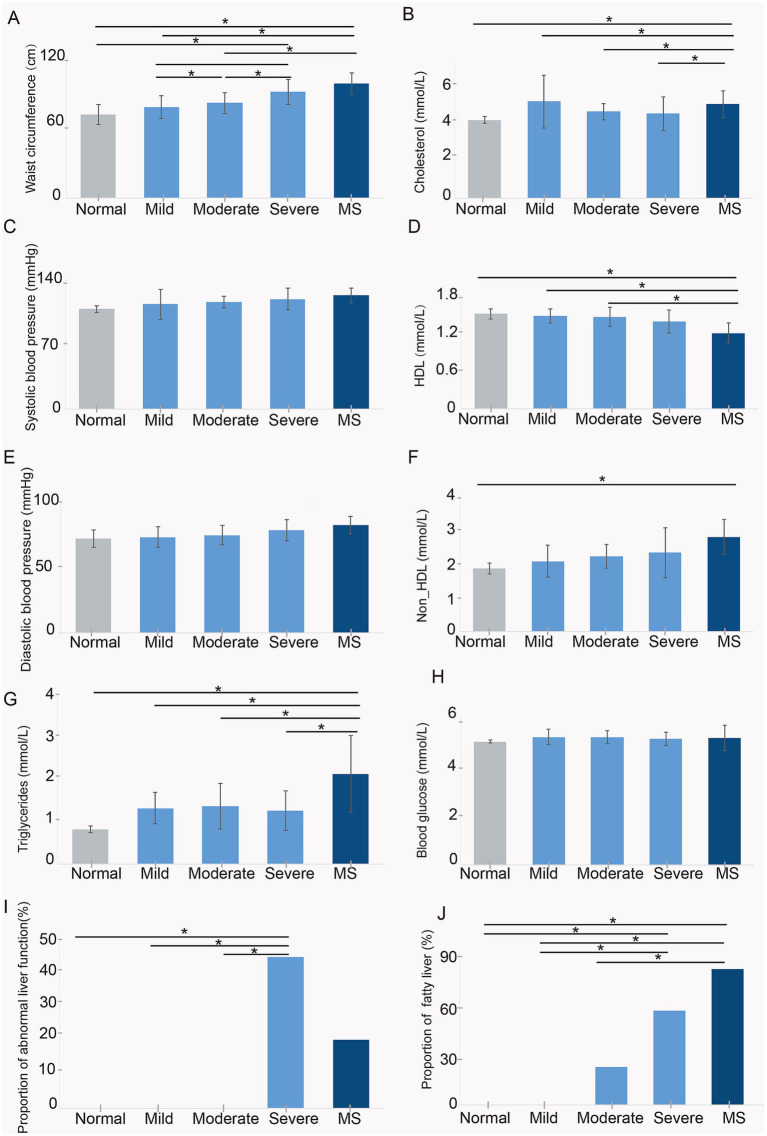
Assessment of physiological and biochemical indicators in five groups of children, including **(A)** waist circumference, **(B)** cholesterol, **(C)** systolic blood pressure, **(D)** HDL, **(E)** diastolic blood pressure, **(F)** non-HDL, **(G)** triglycerides, **(H)** blood glucose, **(I)** proportion of abnormal liver function, and **(J)** proportion of fatty liver. Normal, normal weight; Mild, mild obesity; Moderate, moderate obesity; Severe, severe obesity; MS, metabolic syndrome.

The results indicated no significant differences in blood glucose (Figure 1H), systolic, and diastolic pressure (Figures 1C,E) among the four groups of children. Waist circumference increased with weight, with the highest average in children with metabolic syndrome. Their waist circumference was not significantly different from that of severely obese children but was significantly greater than in the other groups (Figure 1A). Cholesterol and triglycerides were significantly elevated in children with metabolic syndrome compared to others, while children with varying degrees of obesity showed a trend towards higher levels than normal-weight children, though not statistically significant (Figures 1B,G). HDL levels were significantly lower in children with metabolic syndrome compared to normal, mild, and moderate obesity groups, with no significant difference from severely obese children (Figure 1D). Non-HDL levels tended to rise with weight; however, only children with metabolic syndrome had significantly higher Non-HDL levels than normal-weight children, with no significant differences among other obesity groups (Figure 1F). Liver function tests revealed normal function in normal, mild, and moderate obesity children, while 44% of severely obese and 20% of metabolic syndrome children exhibited metabolic dysfunction (Figure 1I), indicating a significant negative impact of obesity on liver health. Furthermore, the prevalence of fatty liver increased with obesity severity, being highest in children with metabolic syndrome and significantly exceeding that of normal, mild, and moderate obesity children (Figure 1J). These findings suggest that childhood obesity, particularly in the context of metabolic syndrome, exerts a substantial adverse effect on physiological and biochemical parameters, notably affecting liver and cardiovascular health.

### Gut microbiota analysis in 32 surveyed children

**Figure 2:**
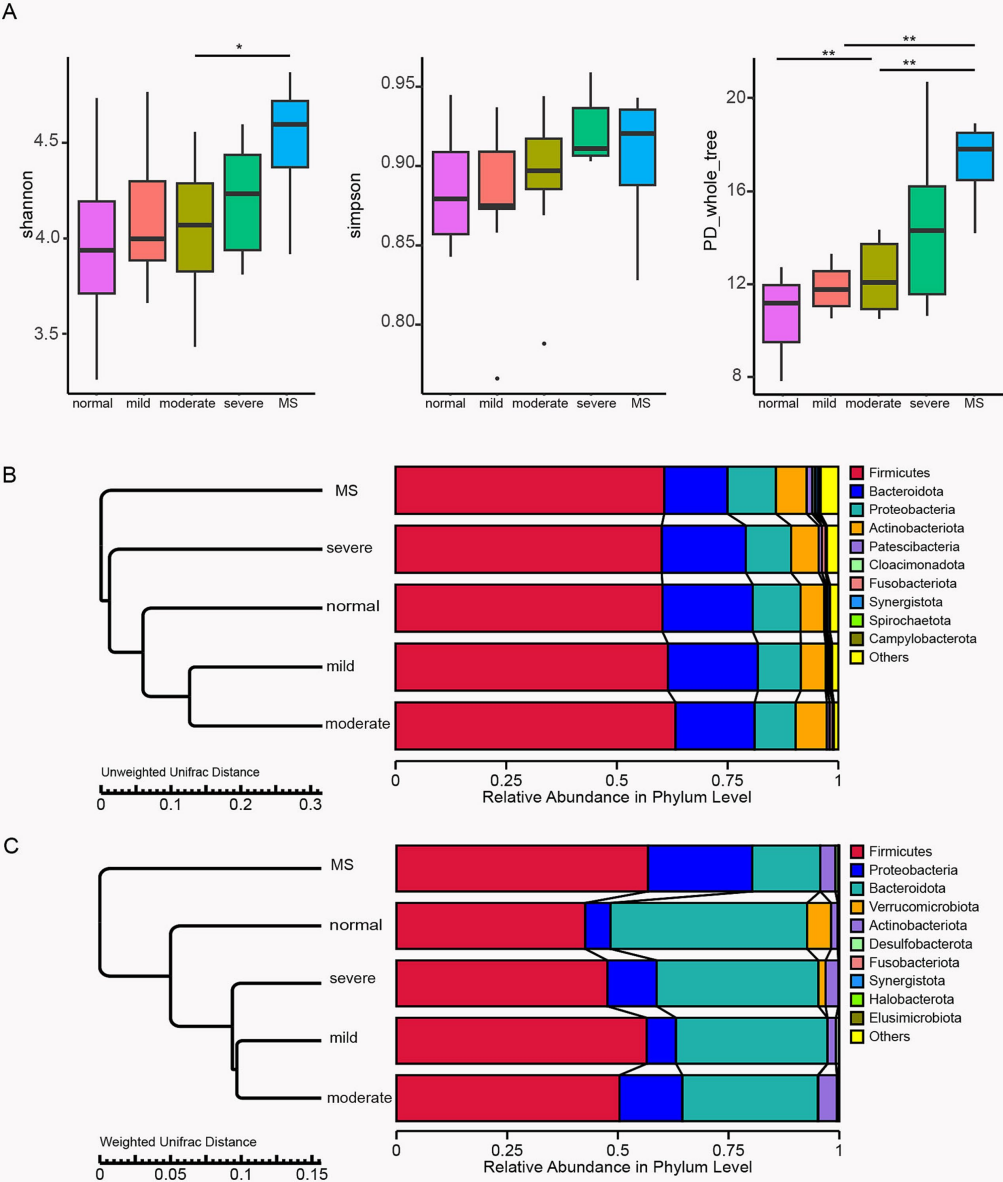
Profiles of gut microbiota in 32 surveyed children. **(A)** Box plots comparing alpha diversity indices, including Shannon, Simpson, and PD_whole_tree indices, among the five study groups. The TTEST was employed to analyze significant differences between the two groups. Bar plots for beta diversity analysis based on relative abundance at the phylum level using Unweighted Unifrac Distance **(B)** and Weighted Unifrac Distance **(C)**. Normal, normal weight; Mild, mild obesity; Moderate, moderate obesity; Severe, severe obesity; MS, metabolic syndrome.

To investigate the impact of gut microbiota on children in our study, we collected stool samples from 32 participants and performed 16S rRNA next-generation sequencing on these samples. After applying strict trimming criteria to obtain high-quality clean tags, the tags were clustered into different OTUs based on similarity. The alpha diversity indices (Shannon, Simpson, and PD_whole_tree) were utilized to evaluate gut microbiota diversity. The results demonstrated that with increasing body weight, all three indices exhibited a positive trend, with children diagnosed with metabolic syndrome showing the highest alpha diversity values (Figure 2A). Specifically, the Shannon index in children with metabolic syndrome was significantly elevated compared to that of moderately obese children, while the PD_whole_tree index was significantly higher in metabolic syndrome group than in those with mild and moderate obesity. Furthermore, the PD_whole_tree index in severe obese children was significantly greater than that of normal-weight children. These findings indicate that body weight significantly affects the alpha diversity of gut microbiota in children, with more pronounced alterations observed in those with metabolic syndrome, reflecting an enhancement in microbial diversity.

In addition to alpha diversity, beta diversity of the stool samples from the five groups of children was analyzed using Unweighted and Weighted Unifrac distances, focusing on the relative abundance at the phylum level of gut microbiota. The analysis using Unweighted Unifrac distances indicated that the gut microbiota composition of children with mild and moderate obesity was closely aligned with that of normal-weight children, while significant divergence was observed in comparison to severely obese and metabolic syndrome children (Figure 2B). In contrast, the Weighted Unifrac distance analysis revealed that the gut microbiota compositions of children across varying obesity levels were relatively similar to those of normal-weight children, with the greatest differences noted in children with metabolic syndrome (Figure 2C). Both Unweighted and Weighted Unifrac distance analyses showed a reduction in the relative abundance of Bacteroidota in obese children, with the lowest proportion found in those with metabolic syndrome, significantly lower than in children with different degrees of obesity. The Weighted Unifrac distance analysis further indicated an increased relative abundance of Proteobacteria in obese children, with a significantly higher proportion in those with metabolic syndrome compared to other obese children. Overall, beta diversity analyses confirm that obesity significantly impacts gut microbiota composition, with more pronounced changes in children with metabolic syndrome, potentially leading to adverse effects on normal metabolic function.

### Taxonomic abundancy difference of normal weight, obese, and metabolic syndrome children

**Figure 3:**
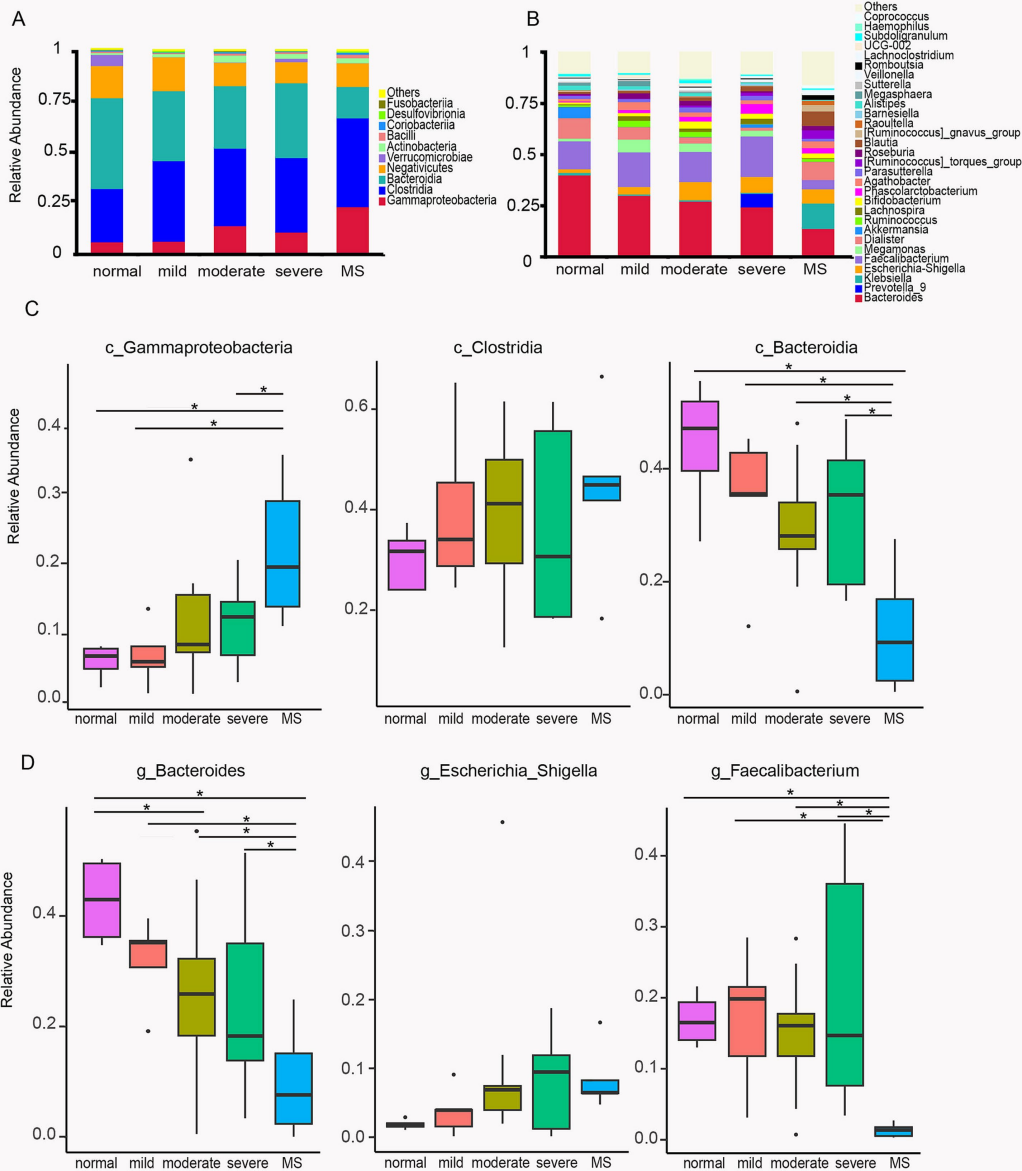
Differences of the community composition of gut microbiota at the class and genus level. **(A)** Profiling of bacterial taxa at the class level. **(B)** Profiling of bacterial taxa at the genus level. **(C)** Statistical analysis of the differences of c_Gammaproteobacteria, c_Clostridia and c_Bacteroidia among the five group. **(D)** Statistical analysis of the differences of g_Bacteroides,g_Escherichia_Shigella and g_Faecalibacterium among the five group. ANOVA analysis with FDR correction was used to analyze significant differences. Normal, normal weight; Mild, mild obesity; Moderate, moderate obesity; Severe, severe obesity; MS, metabolic syndrome.

To further quantify and compare the abundance of each microbial taxon, we first characterized the top ten classes: Gammaproteobacteria, Clostridia, Bacteroidia, Negativicutes, Verrucomicrobiae, Actinobacteria, Bacilli, Coriobacteriia, Desulfovibrionia, and Fusobacteriia (Figure 3A). Gammaproteobacteria and Clostridia exhibited an upward trend in the gut microbiota of children with varying degrees of obesity and metabolic syndrome, while Bacteroidia and Negativicutes showed a downward trend. Statistical analysis indicated that the abundance of Gammaproteobacteria in the gut microbiota of children with metabolic syndrome was significantly higher than that of normal-weight children, as well as in those with mild and severe obesity. Gammaproteobacteria is recognized as a biomarker for overweight, thus corroborating our findings with previous research. Although Clostridia displayed an increasing trend among obese and metabolic syndrome children, statistical analysis did not reveal significant differences across the five groups. The abundance of Bacteroidia in the gut microbiota of children with metabolic syndrome was significantly lower than that in normal-weight children and those with varying degrees of obesity (Figure 3B). Numerous studies have demonstrated that the abundance of Bacteroidia in the gut microbiota of obese populations is markedly reduced compared to normal-weight individuals (Van Hul and Cani, 2023; Wang et al., 2024). Previous research has also indicated a decrease in Bacteroidia abundance among children with metabolic syndrome. Our findings revealed that the abundance of Bacteroidia in the gut microbiota of children with metabolic syndrome was 15.3%, compared to 44.4% in normal-weight children and over 30% in obese children. Consequently, the abundance of Bacteroidia has decreased by nearly half. As one of the most prevalent microbial taxa in the gut, Bacteroidia plays a critical role in human nutrition and immunity, and its substantial reduction will undoubtedly have significant implications for the health of children with metabolic syndrome.

To further investigate the differences in gut microbiota between obese children and those with metabolic syndrome, we conducted a comparative analysis of the gut microbiota composition at the genus level across these five groups of children. In addition to Bacteroides, we observed an upward trend in Escherichia-Shigella in both obese and metabolic syndrome children; however, statistical analysis did not reveal significant differences among the five groups (Figure 3C). Notably, we found that the abundance of Faecalibacterium was significantly reduced in the gut microbiota of children with metabolic syndrome. The abundance in normal-weight children was 13.7%, while in obese children it ranged from 14.7 to 19.7%, and this increase was not statistically significant. In children with metabolic syndrome, the abundance decreased to 4.5% (Figure 3B), representing one-third of the normal levels. Statistical analysis indicated that the abundance of Faecalibacterium in children with metabolic syndrome was significantly different from that in both normal-weight children and those with varying degrees of obesity. Previous studies have reported that a decrease in Faecalibacterium abundance in the gut is strongly associated with elevated inflammatory levels in the body (Martín et al., 2023), suggesting that children with metabolic syndrome experience increased systemic inflammation.

### LEfSe analysis of differential taxa in children with metabolic syndrome and obesity

**Figure 4:**
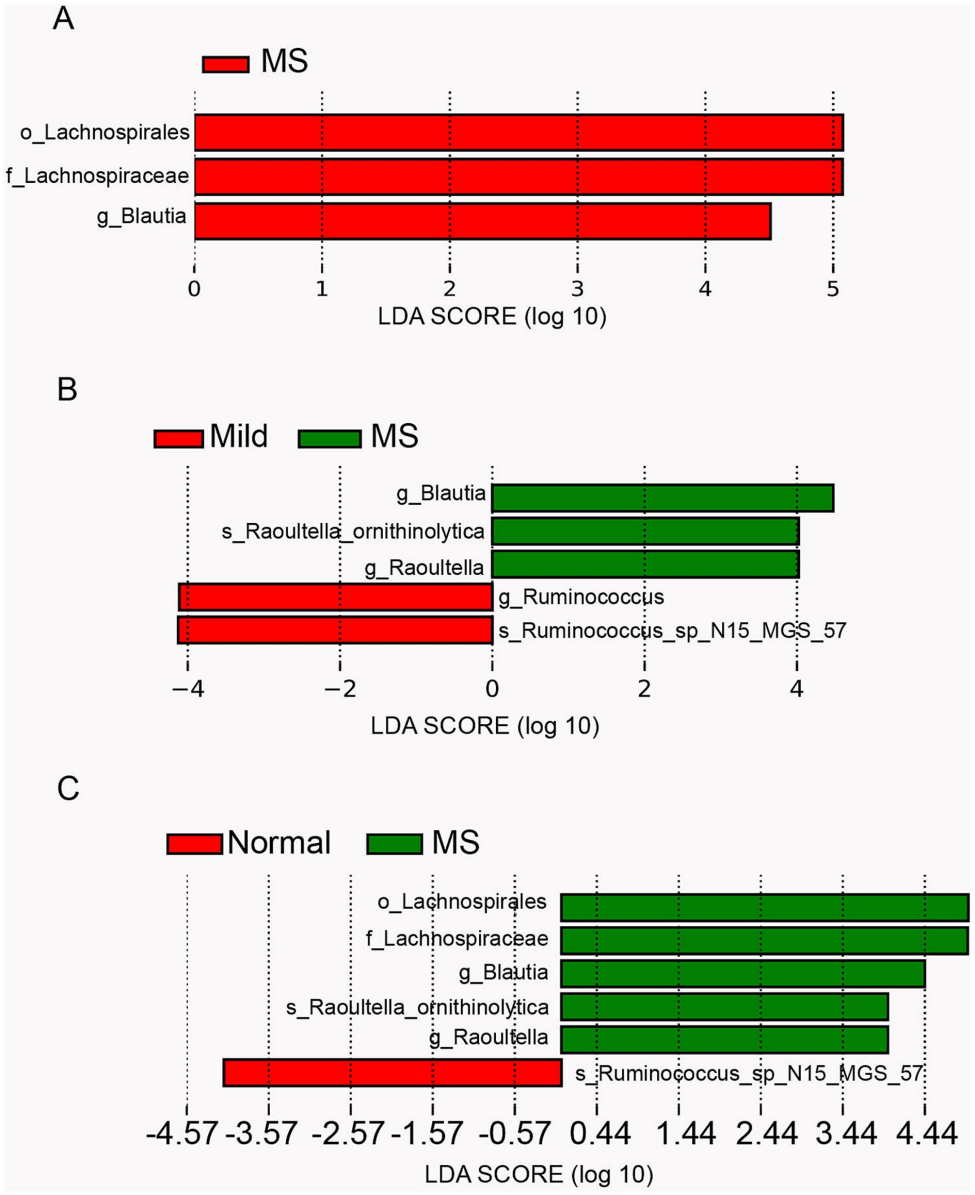
Comparison of gut microbiota abundance using LEfSe analysis. **(A)** Linear discriminant analysis (LDA) for differentially abundant taxa between normal weight and metabolic syndrome groups. **(B)** LDA for differentially abundant taxa among mild obesity, moderate obesity, severe obesity and metabolic syndrome group (a logarithmic linear discriminant analysis score > 3 indicated a higher relative abundance in the corresponding group compared to the other group). **(C)** LDA for differentially abundant taxa among normal weight, mild obesity, moderate obesity, severe obesity and metabolic syndrome group.

Given the similar gut microbiota composition observed in obese children and those with metabolic syndrome, we further confirmed the differentially abundant taxa using LEfSe, a robust algorithm for high-dimensional biomarker discovery. Initially, we utilized LEfSe to identify the differential taxa in the gut microbiota of normal-weight children compared to those with metabolic syndrome. The analysis revealed that the significantly different features (LDA score ≥ 3) included o_Lachnospirales, f_Lachnospiraceae, and g_Blautia, which were exclusively present in children with metabolic syndrome (Figure 4A). Previous studies have indicated that Lachnospiraceae, a member of the Firmicutes phylum, is highly enriched in obese individuals and is associated with insulin resistance, which is closely linked to metabolic syndrome. Furthermore, Blautia has been identified as the only gut microbe significantly and inversely associated with visceral fat area in adults. In contrast, g_Blautia is significantly enriched in the gut microbiota of children with metabolic syndrome, where the prevalence of fatty liver is approximately 80%. Therefore, we hypothesize that the mechanisms underlying the role of Blautia in children may differ from those observed in adults.

Furthermore, we employed LEfSe to identify differential features in the gut microbiota of children with varying degrees of obesity and those with metabolic syndrome. The results revealed that g_Ruminococcus and s_Ruminococcus_sp_N15_MGS_57 were significantly enriched in mildly obese children, while g_Blautia, s_Raoultella_ornithinolytica, and g_Raoultella were significantly enriched in children with metabolic syndrome (Figure 4B). Previous studies have reported that Ruminococcus is associated with obesity in adults, whereas other studies suggest it is linked to leanness in children. Thus, compared to moderately and severely obese children, Ruminococcus was significantly enriched in mildly obese children, suggesting that Ruminococcus may play a role in mitigating obesity in this population. Raoultella is known as an opportunistic pathogen, typically associated with infections of the biliary tract, pneumonia, and bacteremia in oncological patients and those with compromised immunity. The significant enrichment of Raoultella in children with metabolic syndrome implies a potential decline in immune function and the onset of inflammation.

Subsequently, we performed LEfSe analysis on the gut microbiota of normal-weight children, children with varying degrees of obesity, and those with metabolic syndrome. The results were consistent with the comparisons of the previous two groups, revealing significant differences in the gut microbiota between normal-weight children and those with metabolic syndrome. The features significantly enriched in children with metabolic syndrome were the union of those enriched in the above two comparative groups, while s_Ruminococcus_sp_N15_MGS_57 was significantly enriched in normal-weight children. Notably, s_Ruminococcus_sp_N15_MGS_57 was also significantly enriched in children with mild obesity, suggesting a potential association between Ruminococcus and leanness in children (Figure 4C).

### Functional analysis of microbiota in healthy, obese, and metabolic syndrome children

To evaluate the functional disparities in gut microbiota among five cohorts of children, we employed Tax4Fun for comprehensive analysis. The results indicated that the unique functionalities of gut microbiota were as follows: normal children exhibited 0 unique functions, children with mild obesity displayed 7, those with moderate obesity showed 4, severely obese children presented 0, and children with metabolic syndrome demonstrated 81 unique functions (Supplementary Figure S1).

**Figure 5:**
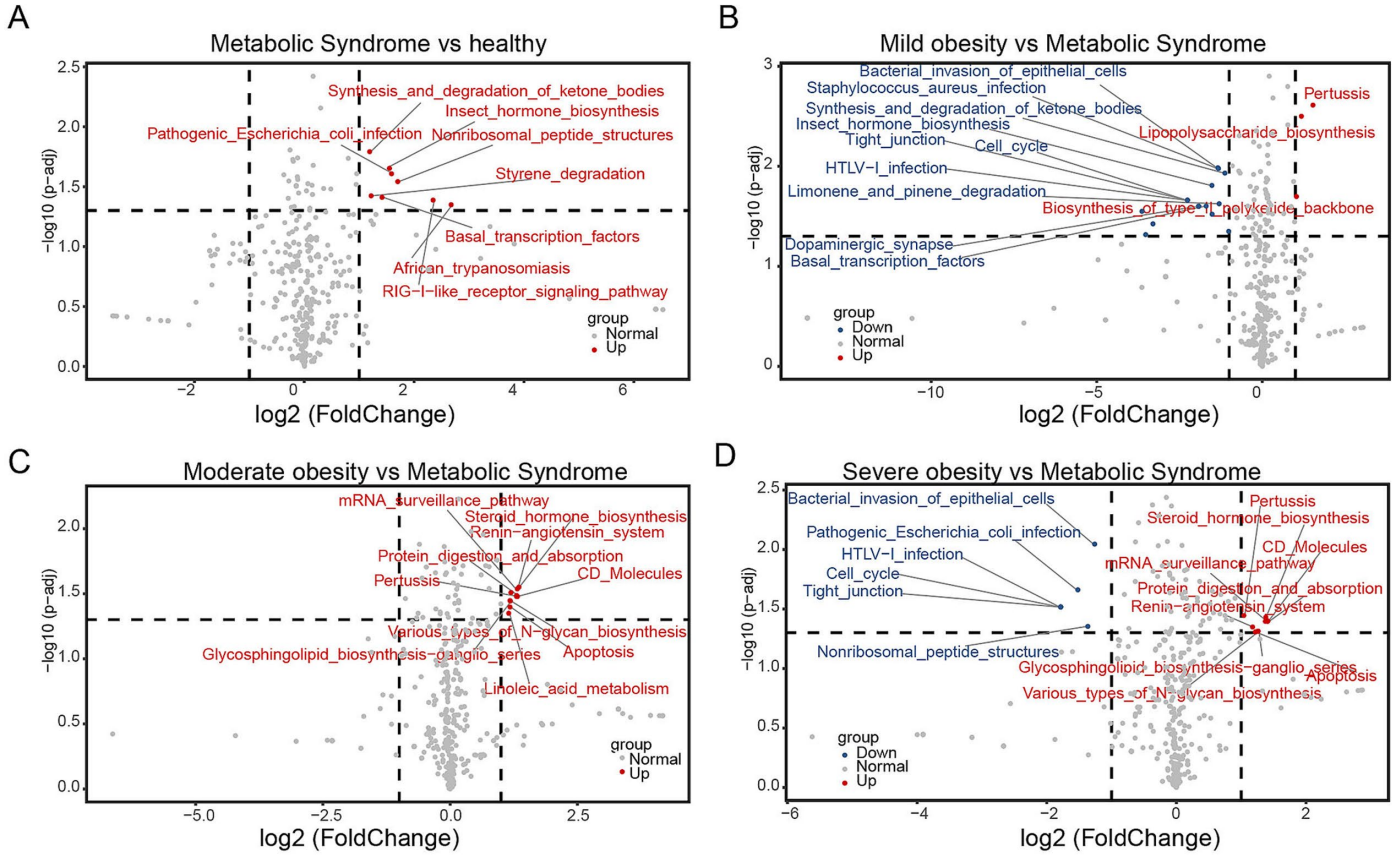
Functional analysis of microbiota between two groups. Volcano plots illustrating the differential functionalities of gut microbiota among children: **(A)** metabolic syndrome versus normal-weight, **(B)** mildly obese versus metabolic syndrome, **(C)** moderately obese versus metabolic syndrome, and **(D)** severely obese versus metabolic syndrome.

To further investigate the characteristics of gut microbiota in children with metabolic syndrome, we conducted a differential analysis of the gut microbiota functions in this group compared to the other four cohorts. The pathway for pathogenic *Escherichia coli* infection was significantly enriched when comparing children with metabolic syndrome to both normal-weight and severely obese children (Figures 5A,D), indicating that children with metabolic syndrome are in a state of bacterial infection, reflecting a suboptimal health status that may lead to various health issues. The Synthesis and degradation of ketone bodies pathway was significantly enriched when comparing metabolic syndrome children to both normal-weight and mildly obese children (Figures 5A,B). Given that obese children typically exhibit insulin resistance, the increased activity of this pathway may reflect metabolic dysregulation, further heightening the risk of metabolic diseases such as diabetes. Comparing children with metabolic syndrome to those with mild and severe obesity, we found significant enrichment in the pathways of Bacterial invasion of epithelial cells, Tight junction, HTLV-I infection, and Cell cycle (Figures 5A,D). The enrichment of Bacterial invasion of epithelial cells and HTLV-I infection indicates bacterial and viral invasiveness within the host, while Tight junction and Cell cycle suggest alterations in intestinal structure and function. Additionally, when compared to moderately and severely obese children, the gut microbiota of children with metabolic syndrome showed significant reductions in Various types of N-glycan biosynthesis, Protein digestion and absorption, and mRNA surveillance pathway (Figures 5C,D), suggesting abnormalities in protein synthesis and utilization. Moreover, we observed a decline in the Steroid hormone biosynthesis function in the gut microbiota of children with metabolic syndrome (Figures 5C,D), potentially affecting various physiological functions such as metabolism, immune response, growth, and development (Melcangi et al., 2002). Importantly, we also found a reduction in the Renin-angiotensin system function (Figures 5C,D), which is primarily involved in blood pressure and fluid balance; an imbalance in this system may adversely affect cardiovascular health. In summary, the functional analysis of gut microbiota indicates that the functionalities of gut microbiota in children with metabolic syndrome have undergone significant changes compared to both normal-weight and obese children, impacting immune responses, glucose-lipid metabolism, protein synthesis and utilization, as well as cardiovascular health.

### Impact of fecal microbiota from children of varying weights on lipid metabolism in intragastric mice

**Figure 6:**
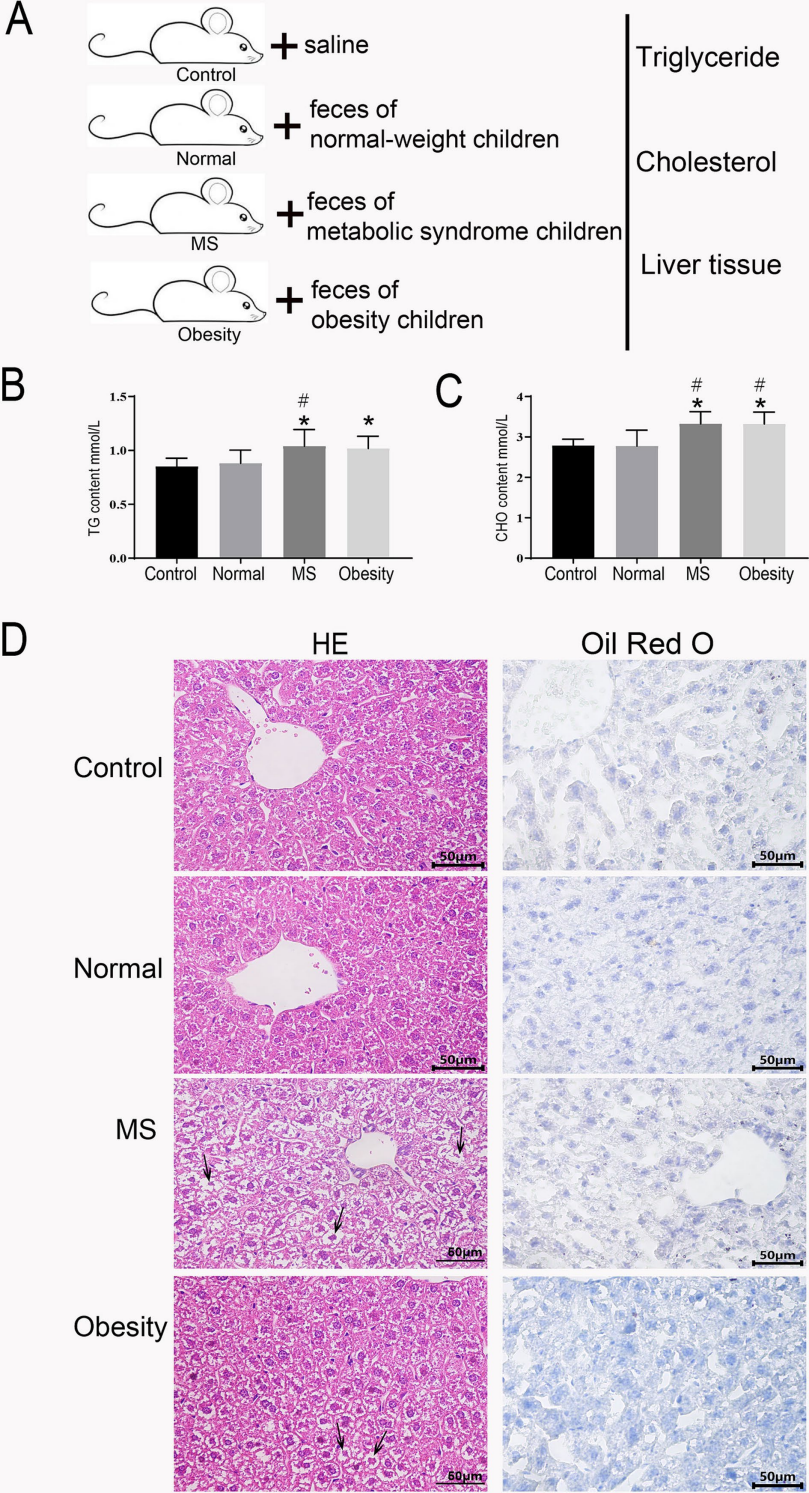
Lipid and liver function assessment in fecal microbiota transplanted mice. **(A)** schematic diagram of the experimental procedure. Serum triglyceride levels **(B)** and Serum cholesterol levels **(C)** in control mice, mice receiving feces from normal-weight children, children with metabolic syndrome, and obese children. **(D)** Histological examination of liver tissue sections from mice receiving feces from normal-weight, metabolic syndrome, and obese children, stained with hematoxylin and eosin (HE) and Oil Red O. Control: Mice gavaged with saline, Normal: Mice gavaged with feces from normal-weight children, MS: Mice gavaged with feces from children with metabolic syndrome, Obesity: Mice gavaged with feces from obese children. * Indicates a significant difference compared to the control group, # indicates a significant difference compared to the normal group. ANOVA analysis with FDR correction was used to analyze significant differences.

To elucidate the significant influence of gut microbiota on lipid metabolism, we conducted a fecal microbiota transplantation experiment utilizing mice maintained under controlled conditions. The mice were stratified into four groups: the first group (Control) received saline via gavage; the second group received feces from normal-weight children (Normal); the third group received feces from children with metabolic syndrome (MS); and the fourth group received feces from obese children (Obesity) (Figure 6A).

After a six-week period, we assessed triglyceride and total cholesterol levels in the serum of the mice. Given the alarming rise in fatty liver prevalence among children with metabolic syndrome (Figure 1J), we also performed HE and Oil Red O staining on liver tissue sections from all four groups. The analysis revealed that the MS group exhibited significantly elevated serum triglyceride levels compared to both the Control and Normal groups (Figure 6B). Similarly, the Obesity group demonstrated significantly higher triglyceride levels than the Control group (Figure 6B). Total cholesterol levels followed a comparable pattern, with both the MS and Obesity groups showing significantly increased serum cholesterol levels compared to the Control and Normal groups (Figure 6C). Oil Red O staining of liver tissue sections revealed substantial nuclear condensation in hepatocytes from the MS group, with no detectable fat accumulation (Figure 6D). The Obesity group exhibited some nuclear condensation as well, yet fat accumulation was absent. In contrast, hepatocytes from the Control and Normal groups displayed clear, intact structures devoid of fat deposits (Figure 6D). The HE and Oil Red O staining results indicated hepatic damage in both the MS and Obesity groups, with the MS group demonstrating more pronounced damage. These findings underscore the significant impact of fecal microbiota transplantation on lipid metabolism in mice, highlighting the critical role of gut microbiota in regulating glucose-lipid metabolism and overall cardiovascular health.

### Impact of normal-weight children’s feces on lipid metabolism in obese mice

**Figure 7:**
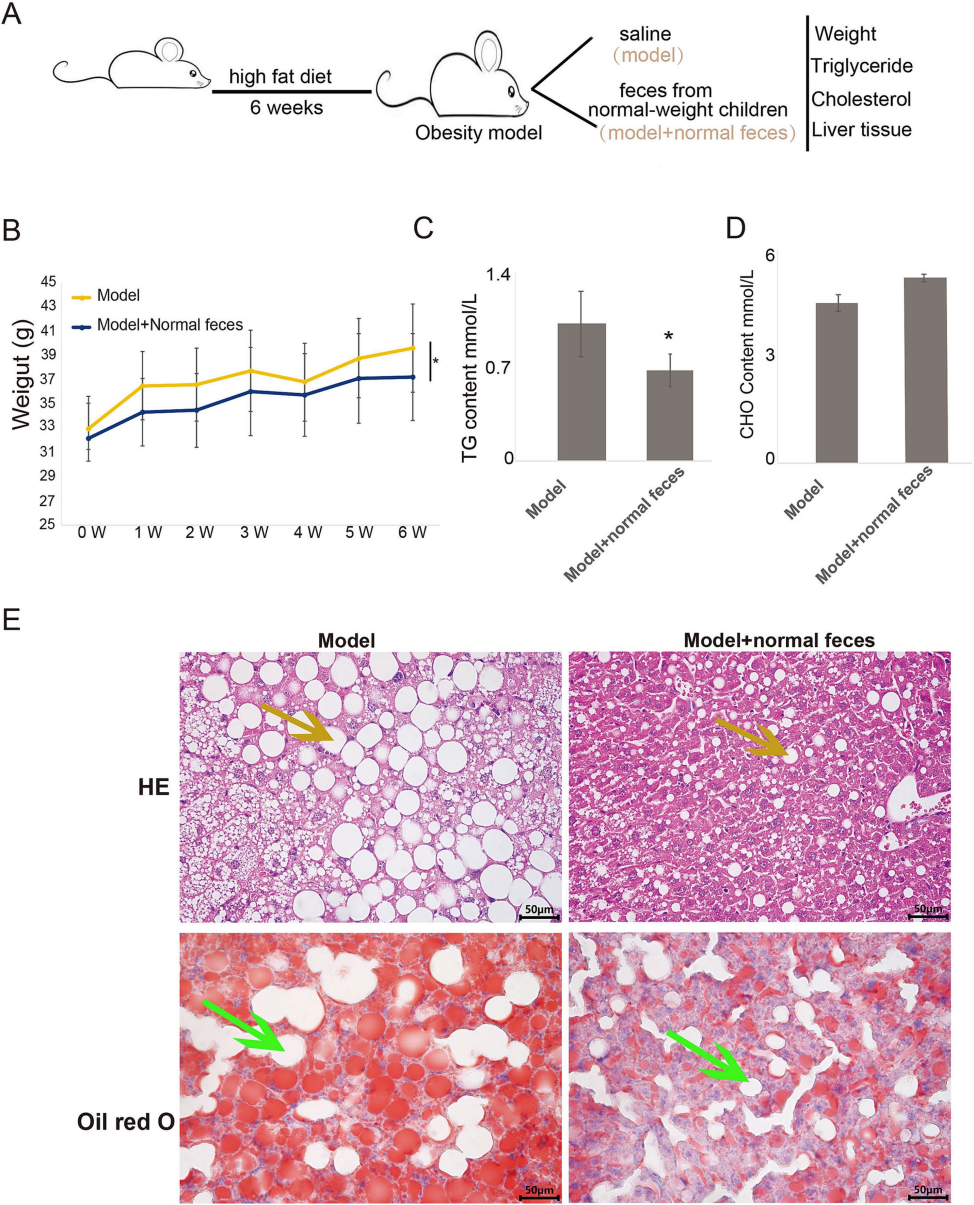
Lipid and liver function assessment in obesity model and fecal microbiota transplanted mice. **(A)** schematic diagram of the experimental procedure. Body weight **(B)**, triglyceride levels **(C)**, and cholesterol levels **(D)** in obese model mice compared to those receiving feces from normal-weight children. **(E)** Histological examination of liver tissue sections from both groups, stained with HE and Oil Red O. W, week; Model, Obesity model mice; Model + Normal Feces, Obese model mice gavaged with feces from normal-weight children. TTEST was used to analyze significant differences. * Indicates a significant difference between two groups.

To further investigate potential interventions for ameliorating lipid metabolism abnormalities in children with metabolic syndrome and obesity, we first established an obesity model using mice. Subsequently, we administered either saline or feces from normal-weight children via gavage to the obese model mice. Following a six-week period, we assessed body weight, serum triglycerides, and cholesterol levels in both groups (Figure 7A).

Our results indicated that, over time, body weight increased in both groups; however, the model mice receiving feces from normal-weight children exhibited significantly lower body weight compared to those receiving saline (Figure 7B). Additionally, serum triglyceride levels in the model mice receiving feces from normal-weight children were markedly reduced compared to their saline counterparts (Figure 7C), while no significant differences in serum cholesterol levels were observed between the two groups (Figure 7D). Histopathological examination using (HE) staining and Oil Red O staining revealed diffuse hepatic steatosis in the model group, characterized primarily by macrovesicular fatty degeneration (indicated by yellow arrows), with extensive fat accumulation evident in the Oil Red O staining (indicated by green arrows) (Figure 7E). In contrast, model mice receiving feces from normal-weight children demonstrated a reduction in fatty vacuoles (yellow arrows) and a minimal presence of fat accumulation as indicated by Oil Red O staining (green arrows) (Figure 7E).

These findings suggest that the gavage of feces from normal-weight children can significantly reduce body weight and improve lipid metabolism in obese model mice. This underscores the potential for probiotic interventions in children with metabolic syndrome and obesity, which may enhance glucose-lipid metabolism, immune function, and cardiovascular health.

**Figure 8:**
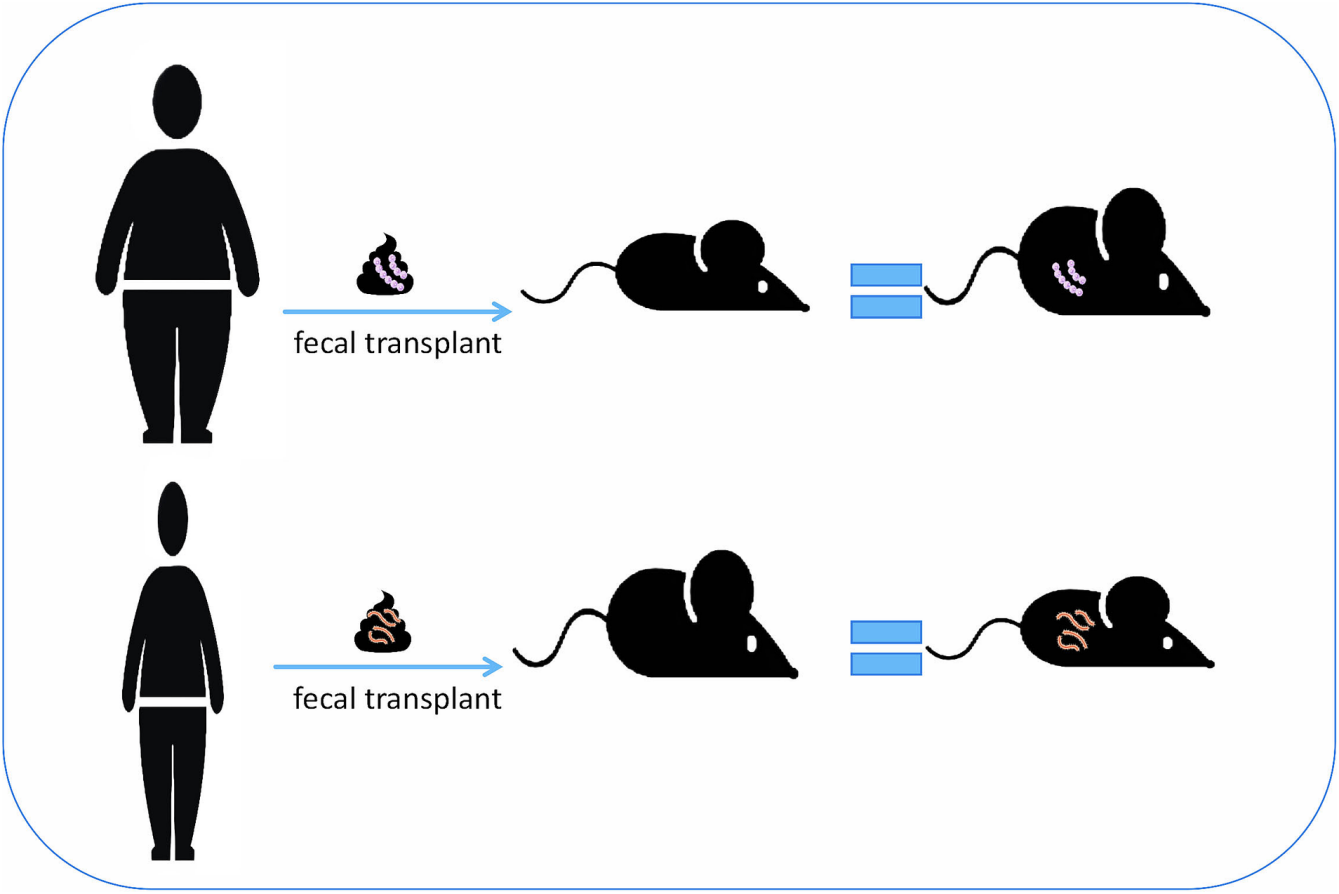
Schematic representation of the influence of gut microbiota on weight regulation in children.

In summary, the transplantation of gut microbiota from children into mice has demonstrated a significant impact on the weight of the mice. This finding suggests that improving the composition of gut microbiota may play a crucial role in alleviating obesity and metabolic syndrome in children (Figure 8). By enhancing the diversity and balance of beneficial microbes in the gut, we may help to create a healthier metabolic environment. This could lead to improved lipid metabolism and overall metabolic health in children, potentially reducing the risk of obesity-related complications. Such insights emphasize the importance of gut microbiota as a target for interventions aimed at promoting healthier weight management in pediatric populations.

## Discussion

In parallel with the rising prevalence of childhood obesity, non-alcoholic fatty liver disease (NAFLD) has emerged as one of the most prevalent health issues among obese children and adolescents (Nobili et al., 2013). Existing data strongly suggest that NAFLD is associated with the key components of metabolic syndrome even in pediatric populations (Selvakumar et al., 2017). Consistent with this, our findings indicate that the prevalence of fatty liver is significantly higher among children with metabolic syndrome compared to those with mild to moderate obesity, showing a trend towards being more prevalent than in severely obese children, albeit without statistical significance (Figure 1J). Numerous studies have explored the potential mechanisms underlying microbiome involvement in NAFLD, including the role of pro-inflammatory bacterial species (Stanislawski et al., 2018) and the production of alcohol (Zhu et al., 2013). Our research demonstrates that the proportion of c_Gammaproteobacteria increases with rising body weight in children, with significantly elevated levels observed in those with metabolic syndrome compared to their obese counterparts. Gammaproteobacteria is implicated in choline metabolism and is found in high abundance among overweight and obese children with NAFLD (Zhao et al., 2019; Michail et al., 2015; Hu et al., 2022). Thus, our study further substantiates the close relationship between gut microbiota and the elevated prevalence of fatty liver in the context of metabolic syndrome.

It has been established that obese individuals exhibit a lower proportion of Bacteroidetes (Clarke et al., 2012). Our study also demonstrated that the proportion of c_Bacteroidia decreases as body weight increases in children (Figure 3C). Notably, we observed a significant reduction in g_Faecalibacterium among children with metabolic syndrome, while no significant differences were found between the proportions in normal-weight and obese children (Figure 3D). Faecalibacterium is known to correlate with inflammatory conditions, particularly inflammatory bowel disease (IBD), and its abundance is diminished in several disorders, including colorectal cancer (CRC), dermatitis, and depression (Martín et al., 2023). This reduction in Faecalibacterium in children with metabolic syndrome may be associated with specific inflammatory states within the gut. Raoultella, recognized as an opportunistic pathogen, is typically linked to infections of the biliary tract, pneumonia, and bacteremia in oncological patients and individuals with compromised immune systems (Sękowska, 2017). LEfSe analysis revealed a significant enrichment of Raoultella in children with metabolic syndrome, suggesting a potential decline in immune function and the onset of inflammation. Functional analysis indicated that the pathway for pathogenic *Escherichia coli* infection was significantly enriched when comparing children with metabolic syndrome to both normal-weight and severely obese children (Figures 5A,D). This finding implies that children with metabolic syndrome may be in a state of bacterial infection, reflecting a suboptimal health status that could predispose them to various health complications.

Furthermore, when comparing children with metabolic syndrome to those with mild and severe obesity, we identified significant enrichment in pathways related to bacterial invasion of epithelial cells, tight junctions, HTLV-I infection, and cell cycle (Figures 5A,D). The enrichment of pathways related to bacterial invasion and HTLV-I infection indicates an increased potential for bacterial and viral invasiveness within the host, while alterations in tight junctions and the cell cycle suggest disruptions in intestinal structure and function. These findings may be linked to the reduced abundance of Faecalibacterium. Consequently, a high proportion of c_Gammaproteobacteria coupled with a low proportion of g_Faecalibacterium could serve as critical biomarkers for the screening of metabolic syndrome.

We collected feces from children of varying weights for intragastric administration to normal mice. Our study demonstrated that the oral administration of feces from obese children with metabolic syndrome to mice resulted in a significant increase in serum triglyceride and cholesterol levels (Figures 6B,C). Histological examination of liver tissue revealed substantial nuclear condensation in hepatocytes (Figure 6D), indicating liver damage. These findings suggest that alterations in gut microbiota have a profound impact on lipid metabolism.

Additionally, We developed obesity model mice and performed intragastric administration of feces from normal-weight children and saline in these mice. Administering feces from normal-weight children to obese model mice led to a reduction in serum body weight (Figure 7B) and triglyceride levels (Figure 7C). Mice receiving feces from normal-weight children exhibited a decrease in fatty vacuoles and fat accumulation in liver (Figure 7E). These results imply that enhancing the gut microbiota in obese children with metabolic syndrome may help to mitigate the abnormal lipid metabolic state and reduce the incidence of fatty liver. Previous studies have indicated that Faecalibacterium has the potential to serve as a next-generation probiotic (NGP) or live biotherapeutic product (LBP) (Martín et al., 2023). Our findings also highlight a significant association between Faecalibacterium and pediatric metabolic syndrome (Figure 3D), suggesting that Faecalibacterium may be a promising candidate strain for the treatment of metabolic syndrome in children.

In summary, our research identified significant differences in both the composition and functional profiles of gut microbiota between children with metabolic syndrome and those with obesity. These findings offer valuable insights into the underlying mechanisms of metabolic syndrome and may inform future diagnostic and therapeutic strategies aimed at managing this condition in pediatric populations.

## Data Availability

The datasets presented in this study can be found in online repositories. The names of the repository/repositories and accession number(s) can be found in the article/Supplementary material.

## Footnotes

^1^ http://www.arb-silva.de/

## Supplementary material

SUPPLEMENTARY FIGURE S1 Unique functionalities of among mild obesity (mild), moderate obesity (moderate), severe obesity (severe) and metabolic syndrome group (MS).
