# Supplementary figures and images for: Comparative analysis of gut microbiota in metabolic syndrome and obese children from Southeastern China
Source: Front Microbiol. 2024 Dec 23;15:1503302. doi: 10.3389/fmicb.2024.1503302 (PMC11701008; doi:10.3389/fmicb.2024.1503302)

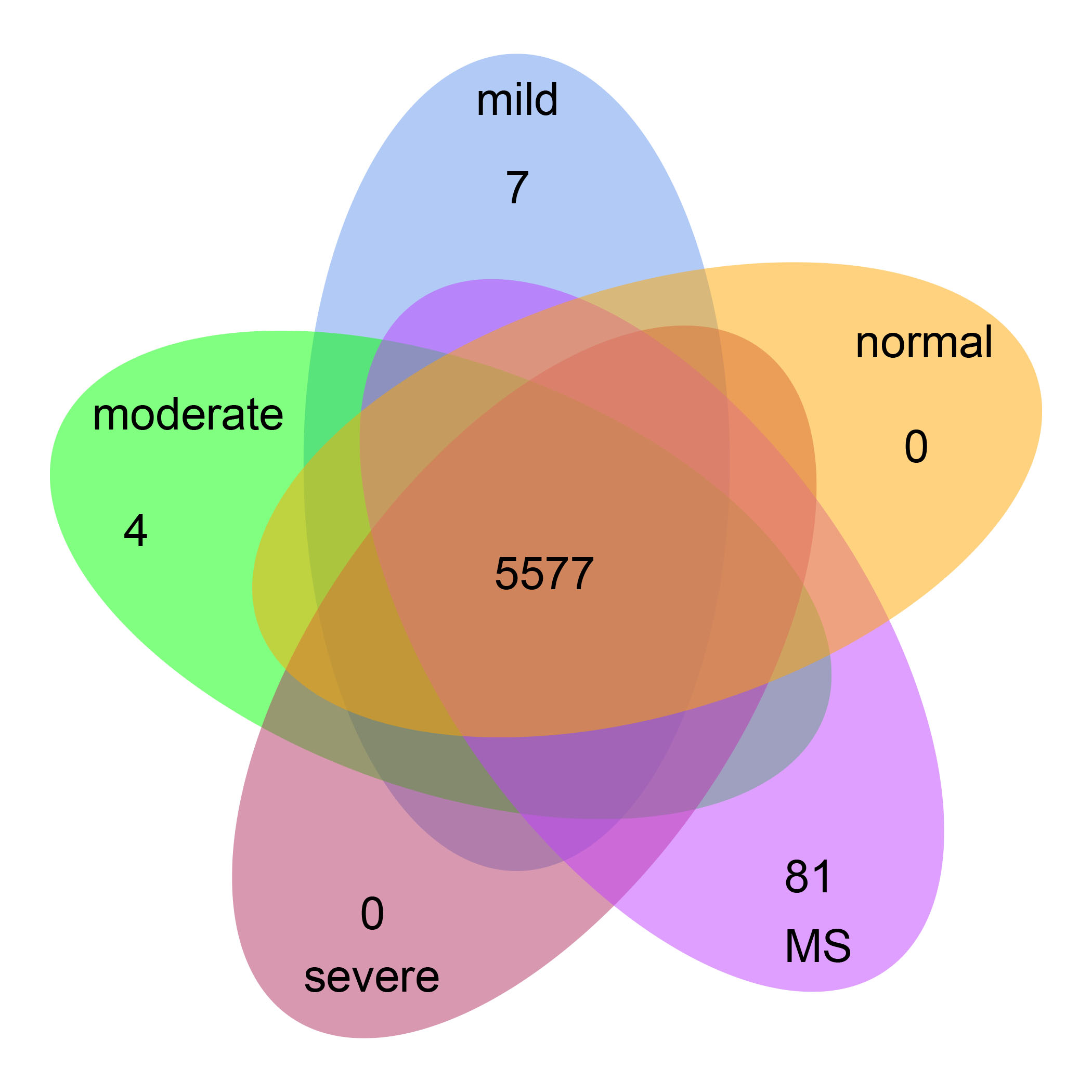

Supplement: SUPPLEMENTARY FIGURE S1 — Unique functionalities of among mild obesity (mild), moderate obesity (moderate), severe obesity (severe) and metabolic syndrome group (MS). [file Image_1.JPEG]
